# Manganese Superoxide Dismutase Gene Expression Is Induced by Nanog and Oct4, Essential Pluripotent Stem Cells’ Transcription Factors

**DOI:** 10.1371/journal.pone.0144336

**Published:** 2015-12-07

**Authors:** Claudia Solari, Camila Vázquez Echegaray, María Soledad Cosentino, María Victoria Petrone, Ariel Waisman, Carlos Luzzani, Marcos Francia, Emilly Villodre, Guido Lenz, Santiago Miriuka, Lino Barañao, Alejandra Guberman

**Affiliations:** 1 Laboratorio de Regulación Génica en Células Madre, Departamento de Química Biológica, Facultad de Ciencias Exactas y Naturales, Universidad de Buenos Aires, Buenos Aires, Argentina; 2 Instituto de Química Biológica (IQUIBICEN), UBA-CONICET, Buenos Aires, Argentina; 3 Laboratório de Sinalização Celular, Departamento de Biofísica, Universidade Federal do Rio Grande do Sul, Porto Alegre, Brasil; 4 Laboratorio de Investigación Aplicada a las Neurociencias (LIAN), Fundación para la Lucha contra las Enfermedades Neurológicas de la Infancia (FLENI), Buenos Aires, Argentina; 5 Consejo Nacional de Investigaciones Científicas y Técnicas (CONICET), Buenos Aires, Argentina; 6 Departamento de Fisiología y Biología Molecular y Celular, Facultad de Ciencias Exactas y Naturales, Universidad de Buenos Aires, Buenos Aires, Argentina; University of Newcastle upon Tyne, UNITED KINGDOM

## Abstract

Pluripotent stem cells possess complex systems that protect them from oxidative stress and ensure genomic stability, vital for their role in development. Even though it has been reported that antioxidant activity diminishes along stem cell differentiation, little is known about the transcriptional regulation of the involved genes. The reported modulation of some of these genes led us to hypothesize that some of them could be regulated by the transcription factors critical for self-renewal and pluripotency in embryonic stem cells (ESCs) and in induced pluripotent stem cells (iPSCs). In this work, we studied the expression profile of multiple genes involved in antioxidant defense systems in both ESCs and iPSCs. We found that Manganese superoxide dismutase gene (Mn-Sod/Sod2) was repressed during diverse differentiation protocols showing an expression pattern similar to Nanog gene. Moreover, Sod2 promoter activity was induced by Oct4 and Nanog when we performed a transactivation assay using two different reporter constructions. Finally, we studied Sod2 gene regulation by modulating the expression of Oct4 and Nanog in ESCs by shRNAs and found that downregulation of any of them reduced Sod2 expression. Our results indicate that pluripotency transcription factors positively modulate Sod2 gene transcription.

## Introduction

Embryonic Stem Cells (ESCs) have a complex network that ensures genomic stability, which is critical as they can give rise to all the cell types of the organism, including the germ line. Their low mutational rate is thought to be a consequence of the concerted action of efficient DNA repair, high fidelity mechanisms, detoxifying activities, and low levels of oxidative stress [1]. Additionally, cells that accumulate mutations are induced to undergo apoptosis or differentiation programs, providing an extra safeguard to the stem cell population genome [2].

Reactive oxygen species (ROS), generated mainly by mitochondrial respiration, play an important role in cellular responses when they are in limited amounts, being second messengers in many signal transduction pathways [3]. Their homeostasis is vital for maintaining several cellular functions such as proliferation, differentiation and apoptosis. Nevertheless, in higher concentration, ROS can modify macromolecules like proteins, lipids and nucleic acids becoming toxic for the cells and even inducing DNA damage [4]. During development, the antioxidant stress defense of the early embryo is challenged by the increasing amounts of ROS resulting in a continuous decrease of glutathione levels [5]. Furthermore, it has been shown that the number of mitochondria and mitochondrial biogenesis is low in ESCs. However, during differentiation mitochondrial proliferation and activity increase [6], concomitantly with an augmented demand for ATP [1,7] and a rise in ROS levels [1]. In addition, there are evidences showing that high levels of ROS promote differentiation in hematopoietic [8,9] and embryonic [10] stem cells.

Induced pluripotent stem cells (iPSCs) have been obtained from multiple cell types since they were developed in 2006 [11]. It has been reported that they have stress defense systems similar to that of ESCs, in spite of being derived from terminally differentiated cells that contain larger amounts of mitochondria. It has also been found that ROS levels in undifferentiated iPSCs are low and that they increase during differentiation, similar to ESC’s [12]. As a whole, these evidences suggest that during the reprogramming process for iPSCs obtention, there is an activation of the cellular mechanisms that provides antioxidant stress defense.

Although it has been shown that mouse and human ESCs express high levels of antioxidant enzymes [1, 6], there is still limited knowledge about their transcriptional regulation. Their modulation and the high complexity of these orchestrated systems led us to hypothesize that some of the genes involved in the cellular oxidative stress defense could be regulated by the transcription factors critical for self-renewal and pluripotency, such as Oct4, Sox2 and Nanog. With the purpose of gaining insight into stress defense factors’ transcriptional regulation, in this work we studied the expression pattern of multiple genes involved in antioxidant defense systems in both ESCs and iPSCs. We found that Manganese superoxide dismutase gene (Mn-Sod/Sod2) was highly expressed in pluripotent stem cells and repressed during differentiation and that its promoter was induced by the pluripotency transcription factors Oct4 and Nanog.

## Materials and Methods

### Cell culture, culture conditions and differentiation

R1 and Ainv15 ESC lines are commercial lines purchased from ATCC. They were cultured and differentiated as previously described [13–15]. The iPSC-20 line was derived and validated previously by us [16], and cultured and differentiated as previously described [16]. This line was obtained by reprogramming day 13.5 mouse embryonic fibroblasts transduced with pHAGE-EF1a-STEMCCA [17]. NIH/3T3 cell line (ATCC) was cultured in DMEM supplemented with 10% FBS (Gibco) and antibiotics [100 U/ml penicillin and 100 mg/ml streptomycin (Gibco)]. 46C Sox1-GFP ESC line [18] was a kind gift from Austin Smith and were cultured in 0.1% gelatin coated plates in N2B27 2i/LIF medium, composed of serum free N2B27 based medium [19] (prepared using B27 without Vitamin A) and supplemented with 0.1 mM 2-mercaptoethanol, 10 μg/ml human insulin (Sigma), Glutamax, 1 μM MEK Inhibitor PD0325901 (Tocris), 3 μM GSK3 inhibitor CHIR99021 (Tocris) and 1000 units/ml of LIF (ESGRO). To induce neural progenitor differentiation, cells were cultured for 24 h at high density conditions (1x10^5^ cells/cm^2^) in N2B27 2i/LIF medium, which maintains the cells in undifferentiated state. On the following day, cells were harvested and resuspended in N2B27 medium with Vitamin A-containing B27 (N2B27-RA), and plated at 1x10^4^ cells/cm^2^ onto Fibronectin (Sigma) coated dishes. Differentiation N2B27 base medium was prepared using DMEM:F12 and Neurobasal media without phenol red. Cells were cultured until day 6 with change of medium every other day. Efficacy of the differentiation protocol was visualized by fluorescence microscopy.

### Quantitative real time RT-PCR (RT-qPCR)

Total cellular RNA was isolated from subconfluent cultures or EBs using TRIzol reagent according to the manufacturer’s instructions (Life Technologies). The yield and purity of RNA samples were assessed by the absorbance at 260 nm and 260 nm/280 nm ratio, respectively. One μg of total RNA was retro-transcribed using MMLV reverse transcriptase (Thermo Scientific) and Random Primers (Invitrogen) according to the manufacturer’s instructions. Quantitative Real time PCR amplification of DNA was carried out using FastStart SYBR Green Master (Roche) and specific oligonucleotides (S1 Table) in Opticon Real Time DNA engine (Bio-Rad). A melting curve analysis was performed immediately after amplification at a linear temperature transition rate of 0.2°C/s from 61°C to 91°C with continuous fluorescence acquisition. The amplicon size was confirmed by gel electrophoresis. Raw data were analyzed with LinReg PCR software and N_0_ fluorescence values were calculated using the same program. Gene expression was normalized to the housekeeping gene Glyceraldehyde 3-phosphate dehydrogenase (*Gapdh) or to the geometrical mean of Gapdh and Phosphoglycerate kinase 1 (Pgk1) expression and referred to the control condition*, *as indicated in each case*. A no-template blank served as negative control.

### Cloning and construction of reporter vectors

To construct the reporter vectors pSod2.1-Luc and pSod2.2-Luc, a 1142 bp fragment and a 1533 bp fragment of the promoter region of Sod2 were amplified by PCR from R1 ESCs genomic DNA, respectively, and they were cloned into MluI and XhoI cloning sites in the pGL3-Basic vector (Promega) upstream of the Luciferase gene. The oligonucleotides are listed in S1 Table. Restriction enzymes were obtained from Promega. All constructs were verified by DNA sequencing.

### Transfection and luciferase activity assay

NIH/3T3 cells were co-transfected in 24-well plate with 300 ng of pSod2.1-Luc or pSod2.2-Luc reporters and 0, 100, 200 or 400 ng of pMXs-Nanog and/or pMXs-Oct4 (Addgene). Transfection was carried out using PEI (Linear Polyethylenimine 25 kDa, Polysciences, Inc.) with a DNA/PEI ratio of 1:3. For normalization of transfection efficiency, 20 ng of pRL-TK reporter (Promega), constitutively expressing the *Renilla reniformis* luciferase, was included in each transfection assay. After ON incubation, the medium was replaced by fresh medium. After 24 h, cells were lysed and assayed for luciferase activity using the Dual Luciferase kit (Promega) on a GloMax Multi Detection System (Promega). Experiments were performed in triplicate and repeated at least three times.

### Downregulation of transcription factors by shRNA approach

R1 ESCs cultured in standard medium on gelatin coated p60 plates, were transfected with 3 μg pLKO.1-puro derived vectors (Sigma), expressing shRNA targeting Nanog (SHCLND-XM_132755), Oct4 (SHCLND-NM_013633) or eGFP (SHC005), which was used as control vector. Transfection was carried out using PEI, as transfection reagent, with a DNA/PEI ratio of 1:5. After ON incubation, the medium was replaced by fresh medium. Twenty four hours after transfection, transfected cells were selected for 48 h with puromycin at 3 μg/ml final concentration. Then, total RNA was isolated using TRIzol reagent (Life Technologies) and mRNA expression was analyzed by RT-qPCR as described in the RT-qPCR section.

### Statistics and data analysis

**Fig 1:**
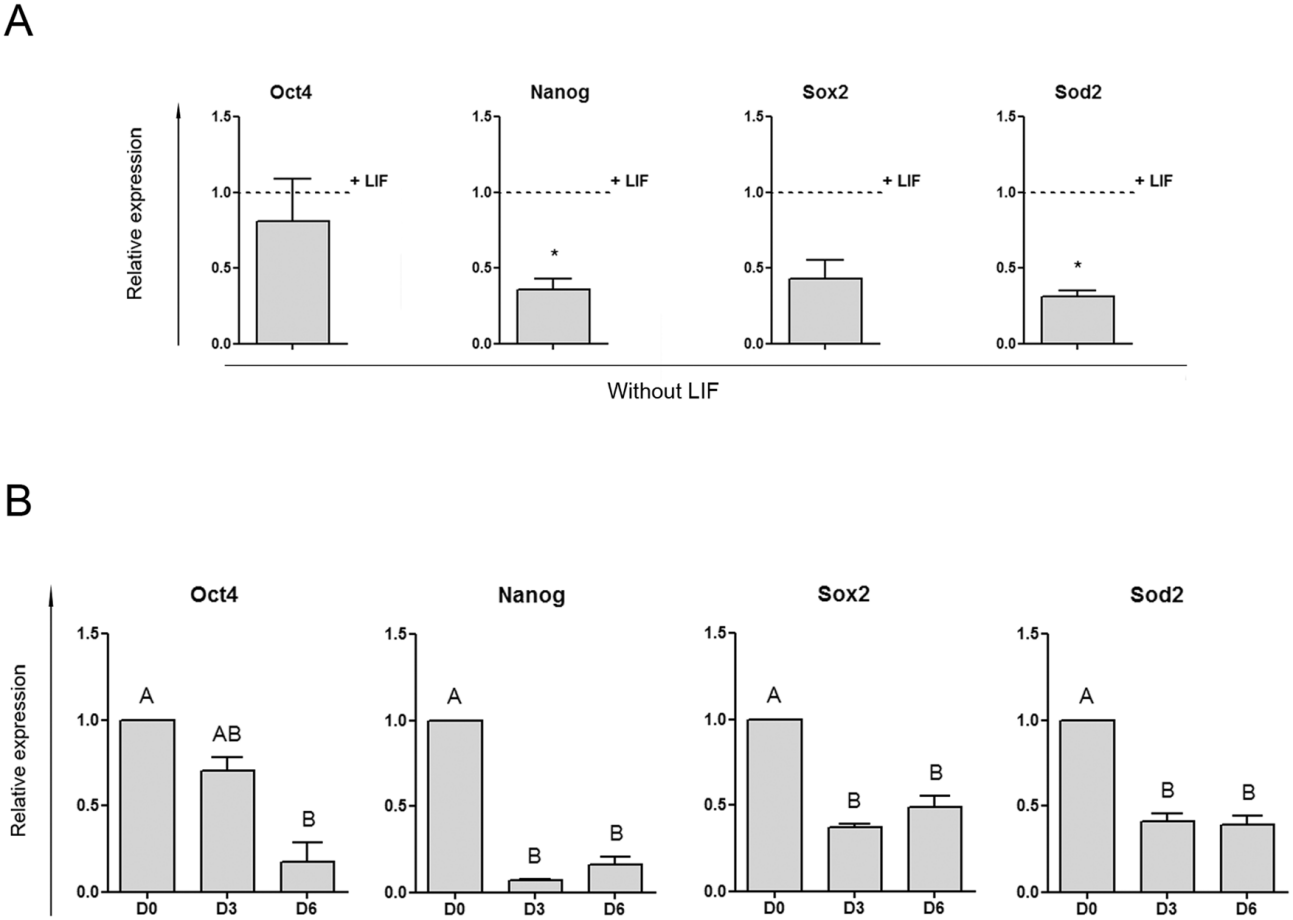
Sod2 is repressed in ESCs subjected to distinct differentiation protocols. ESCs were cultured as described in each case. Then, RNA was extracted and the expression of the indicated genes was measured by RT-qPCR. Gene expression was normalized to the geometrical mean of Gapdh and Pgk1 expression and referred to the control condition. Results are shown as mean ± SEM of three independent experiments. **(A)** R1 ESCs were cultured under standard conditions in the presence of LIF (control, shown as a dashed line) or in the absence of LIF, for 4 days. * p < 0.05. **(B)** 46C ESCs were subjected to a neural progenitor differentiation protocol. Expression of the indicated genes was analyzed at days 0 (D0, control), 3 (D3) and 6 (D6) after the induction of differentiation. Different letters (A or B) indicate statistically significant differences between treatments. AB indicates no statistically significant difference either to A or to B (p < 0.05).

**Fig 2:**
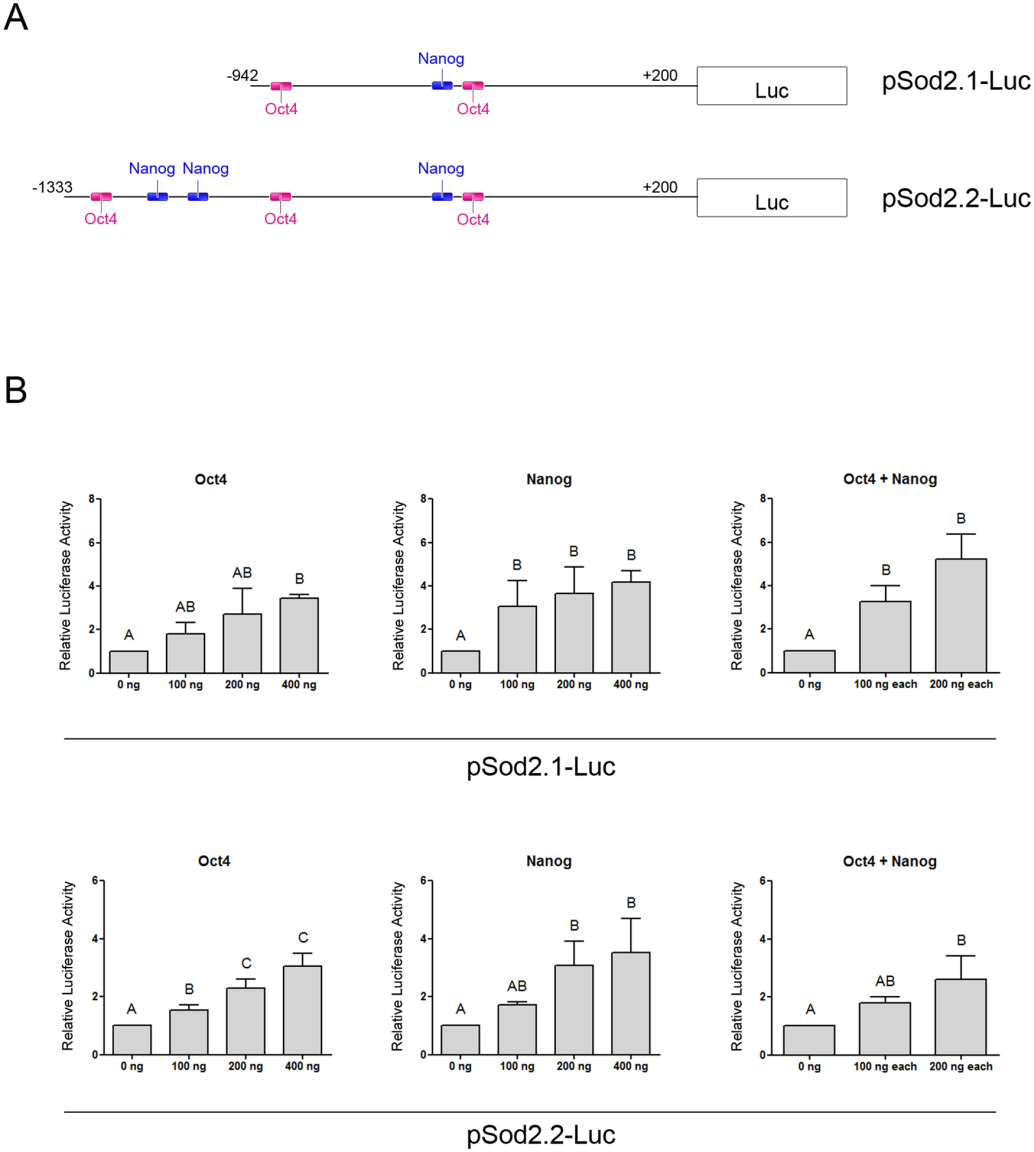
Oct4 and Nanog induce pSod2-Luc constructions. **(A)** Scheme of pSod2.1-Luc and pSod2.2-Luc constructions showing the putative binding sites for Oct4 and Nanog. **(B)** NIH/3T3 cells were transfected with pSod2.1-Luc or pSod2.2-Luc and with the indicated amounts of pMXs-Nanog, pMXs-Oct4 or both. Luciferase activities were measured as described in Material and Methods. Values were normalized to *Renilla*’s luciferase and referred to the basal condition (without the addition of any transcription factor). Results are shown as mean ± SEM of at least three independent experiments. Different letters indicate statistically significant differences between treatments (p < 0.05).

Experimental results are presented as mean ± standard error of the mean (SEM). Statistical comparisons were performed using randomized block design ANOVA for biological replicates using Infostat statistical software [20]. For analysis of the experiments shown in Figs 1, 2 and S2 Fig, data were transformed with log_10_. Residuals fitted normal distribution and homogeneity of variance. When necessary, Tukey Test was used for comparisons between means. p values < 0.05 were considered significant.

## Results

Based on previously reported evidences, we decided to look for genes involved in the response to oxidative stress, which might have a differential expression pattern in undifferentiated stem cells compared to differentiated cells. We hypothesized that genes that are modulated during differentiation could be transcriptionally regulated by pluripotent stem cells’ specific transcription factors. To achieve this, we first analyzed *in silico* the promoter regions of multiple genes that encoded different proteins or enzymes involved in redox metabolism, such as Catalase (Cat), Glutaredoxin (Glrx), Glutathione peroxidases (Gpx), Glutathione reductase (Gsr), Peroxiredoxins (Prdx), Superoxide dismutases (Sod), Thioredoxins (Txn), and Thioredoxin reductases (Txnrd). We searched for predicted binding sites for transcriptions factors expressed in pluripotent stem cells that are critical for self-renewal and pluripotency, particularly for Oct4, Sox2 and Nanog. As shown in S2 Table, all the analyzed sequences from the selected genes contained putative binding sites for at least one of the transcription factors. The position of each predicted site is detailed in S3 Table.

To identify genes that are differentially expressed in undifferentiated compared to differentiated state, we studied their expression in three pluripotent stem cell lines: two ESC lines and an iPSC line generated by us, iPSC-20 [16]. We used two different mouse ESC lines, R1 and Ainv15, which were derived from embryos of different strains. Since genetic background influences gene expression, we reasoned that obtaining similar results in different cell lines could lead us to find out conserved mechanisms, extending our results. With the purpose of analyzing gene modulation along the differentiation process, we generated embryoid bodies (EBs) using an *in vitro* differentiation protocol, both for ESCs and iPSCs and analyzed the selected genes’ mRNA levels by quantitative RT-PCR (RT-qPCR), from days 0, 4 and 7 after plate attachment. Concomitantly, we measured the expression of the pluripotency gene markers Oct4, Nanog and Sox2, to confirm the differentiation protocol. We found a great diversity in the transcriptional profile of the selected genes, some being upregulated along the process, others highly repressed, and some resulted unaffected. Moreover, we observed high variability in almost all analyzed genes, among the different replicates even within the same cell line (S1 Fig), probably as consequence of the non-directed nature of the hanging drop differentiation. Although we could not find a defined profile in most of the evaluated genes, we systematically found that Sod2 gene was repressed along the differentiation in the three studied cell lines.

Based on the results of our screening and in the vast evidence that reports the high relevance of Sod2 gene in oxidative stress cellular defense, we decided to further investigate its modulation. For this purpose, we next compared ESCs cultured in standard culture medium in the presence of LIF (control) with cells cultured in the absence of this cytokine for 4 days, a condition that drives cells out from the pluripotent state. As shown in Fig 1A, both Sod2 and Nanog gene expression showed a significant decrease in the absence of LIF. Finally, we also found Sod2 repression during a neural progenitor differentiation protocol performed in an ESC reporter line that expresses GFP driven by Sox1 promoter, a specific marker of neuroectoderm [18] (Fig 1B and S2 Fig).

Taking into account the similar behavior between Sod2 and Nanog expression observed in the studied differentiation protocols and on the presence of putative consensus sites for pluripotency transcription factors in Sod2 promoter, we decided to study whether pluripotent stem cells’ transcription factors regulate Sod2 gene expression in a more defined system. To achieve this, we constructed two reporter vectors containing fragments from the promoter region of this gene cloned upstream from the firefly luciferase reporter gene. We generated pSod2.1-Luc reporter vector containing a fragment from -942 to +200 respect to the transcription start site of the promoter region of Sod2 driving the reporter gene, and pSod2.2-Luc, containing a larger fragment from -1333 to +200. The first fragment contains three putative binding sites for stem cells’ transcription factors, two for Oct4 and one for Nanog; the larger fragment contains one more site for Oct4 and two more sites for Nanog (Fig 2A). For performing the transactivation assay, we chose the NIH/3T3 mouse embryonic fibroblast cell line, in which we could not detect Oct4 or Nanog mRNA levels. The reporter constructs were co-transfected with different amounts of the expression vectors for Nanog, Oct4 or both. As outlined in Fig 2B, although we didn’t find a synergic effect, both transcription factors were capable of significantly induce luciferase expression in a dose-dependent manner both in pSod2.1-Luc and in pSod2.2-Luc. These results indicated that the selected Sod2 promoter regions are induced by both Oct4 and Nanog.

**Fig 3:**
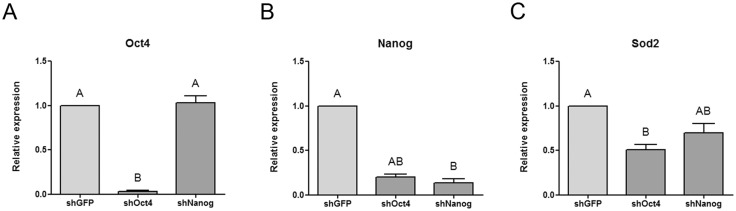
Sod2 is repressed in R1 ESCs transfected with shRNA targeting Oct4 or Nanog. R1 ESCs were transfected with pLKO.1-puro derived vectors targeting stem cells’ transcription factors (shOct4 or shNanog), or eGFP (shGFP, control), as indicated under each bar. Then, transfected cells were selected with puromycin for 48 hs and RNA was extracted. The expression levels of Oct4 **(A)**, Nanog **(B)** or Sod2 **(C)** were analyzed by RT-qPCR and referred to the control. Gene expression was normalized to the geometrical mean of Gapdh and Pgk1 expression and referred to the control condition. Results are shown as mean ± SEM of at least four independent experiments. Different letters indicate statistically significant differences between treatments (p < 0.05).

Next, to study the effect of Oct4 and Nanog transcription factors on the expression of the endogenous Sod2 gene, we downregulated their expression by a shRNA approach. We transfected R1 ESCs with vectors expressing shRNA targeting Nanog (shNanog), Oct4 (shOct4) or eGFP (shGFP) as a control, and then analyzed Oct4, Nanog and Sod2 gene expression by RT-qPCR. As shown in Fig 3, both key transcription factors were downregulated by their specific shRNA. Moreover, Nanog was also repressed in R1 ESCs transfected with shOct4, as expected based on previous reports [21]. We then analyzed Sod2 mRNA levels and found a reduction of about 35% when Nanog was downregulated and about 50% in ESCs transfected with the shOct4. These results are in agreement with the fact that Sod2 gene was expressed in pluripotent stem cells and repressed along differentiation, and with the aforementioned transactivation assay findings, indicating that both Oct4 and Nanog, critical transcription factors to maintain the pluripotent state, positively modulate Sod2 gene transcription.

## Discussion

ROS generated by mitochondrial respiration play an important role in maintaining cellular functions. During embryo development, changes in metabolism take place shifting from glycolysis to oxidative phosphorylation [3]. Concomitantly, somatic cells switch their metabolism from an oxidative to a glycolytic state when they are reprogrammed and these changes are critical to generate iPSCs quicker and more efficiently [22]. The production of ROS as natural by-products of metabolism is a consequence of using oxygen as an electron acceptor, and when an imbalance in the redox homeostasis occurs, ROS are a considerable cause of DNA damage. Both ESCs and iPSCs have complex and coordinated mechanisms that ensure the maintenance of their genomic stability. This network is composed by multiple enzymes and non-catalytic proteins that work together to preserve an accurate redox balance. Although these components have been extensively studied for many years, and it was reported that antioxidant defense activity diminishes during differentiation [1,7], little is known about the mechanisms involved in their transcriptional regulation.

In this work, we studied the gene expression pattern of some of the components of the oxidative stress defense system in ESCs and iPSCs in the undifferentiated state and during differentiation. We selected and analyzed a group of genes under the hypothesis that some of these are transcriptionally regulated by the transcription factors critical for stem cells pluripotency.

We first selected relevant proteins from different functional groups belonging to the antioxidant defense system. We decided to study the glutathione/thioredoxin system since glutathione is the main redox buffer for thiol-disulfide groups of the cell [23]. This system and others related to oxidative stress defense involve multiple genes and several of them were shown to be modulated during differentiation [1,7,12,24]. For example, it has been reported that Manganese superoxide dismutase (Mn-Sod/Sod2) and some glutathione peroxidases are repressed during mouse ESCs differentiation [7]. Furthermore, the same modulation was found for Sod2 and Gpx2 in human ESCs [1]. Sod2 is a mitochondrial enzyme that converts superoxide anion into peroxide, which is substrate for Prdxs and Gpxs that produce H_2_O as a final product. On the other hand, glutathione reductase, that restores reduced glutathione that was oxidized by Gpx, was also reported to decrease its expression along differentiation, both in human ESCs and iPSCs [1,24]. However, there is a report that differs from those previously mentioned, where the expression level of proteins was analyzed instead of the mRNAs, showed a subtle variation in Sod2 expression and a decreased expression of Catalase during human ESCs differentiation [22]. Furthermore, it was found that the thioredoxin inhibitor, Thioredoxin-interacting protein (TXNIP), increased its expression during differentiation [1]. We were interested in studying the behavior of other enzymes involved in the defense system such as Thioredoxin 1, that is essential in embryogenesis and its absence does not allow the proliferation of the inner cell mass [25]; Glutaredoxin, critical for cell cycle progress during embryogenesis [26]; and finally Thioredoxin 2 and Thioredoxin reductases, also proved to be relevant to embryogenesis [27,28].

Based on these evidences, we selected a group of fourteen genes and analyzed their promoter regions searching for putative binding sites for the transcription factors Oct4, Nanog and Sox2. We next investigated the expression profile of the selected genes along a hanging drop differentiation protocol compared to undifferentiated cells. We reasoned that genes whose modulation could be detected among the high diversity of cell types present in embryoid bodies in a non-directed differentiation could play a relevant role in the undifferentiated state. Many of the selected genes did not change or did not show a similar pattern to Oct4, Sox2 or Nanog. However, we found that Gsr, Sod1 and Sod2 were consistently downregulated during the differentiation processes in both R1 ESCs and iPSCs. In this study, we focused on Sod2 modulation by Oct4 and Nanog.

Sod2 is the member of superoxide dismutases family whose knock out in mouse has the most severe phenotype [29,30], suggesting that the toxicity of the mitochondrial ROS is highly deleterious. Moreover, Sod2 deficient mice display multiple biochemical features of mitochondrial disease associated with ROS toxicity [31]. Regarding its regulation, bioinformatic analyses have revealed many transcriptional regulatory elements in the proximal promoter regions of all three Sod genes that are putative binding sites for several common transcription factors [32]. However, to our knowledge there are no reports about the effect of pluripotency transcription factors on Sod2 gene modulation. This gene has been previously mentioned in a genome wide analysis among other genes whose expression decreased after LIF starvation in mouse ESCs [21]. It was also reported to be modulated in a genome wide meta-analysis applied to multiple gene expression datasets from three mouse ESCs lines during differentiation into various lineages [33]. Recently, it was reported that Sod2 promoter was induced by LIF in a JAK2/STAT3 dependent manner, and that Sod2 silencing resulted in the loss of pluripotency, even in presence of LIF. Moreover, the authors showed that Sod2 overexpression was sufficient for maintaining the expression of pluripotent genes in the absence of STAT3 signaling in R1 ESCs. As a whole, they propose that Sod2 may also play a role in mouse ESCs pluripotency besides its known function as a regulator of ROS levels [34]. It has also been previously reported that STAT3 induced Sod2 transcription in mouse normal brain but it was downregulated after reperfusion in mouse cerebral ischemic injury with a concomitant increase in superoxide anions [35].

In this work we found that Sod2 expression pattern was similar to Nanog’s, one of the main pluripotency transcription factors in pluripotent stem cells, when these cells were subjected to three distinct differentiating conditions, two non-directed protocols, the hanging drop protocol and the culture in absence of LIF, and a directed neural precursor differentiation protocol. As a whole, distinct differentiating contexts led us to find out that Sod2 gene was repressed when ESCs leave behind the undifferentiated state. Next, using a transactivation assay, we found that both Oct4 and Nanog induced two different fragments from Sod2 gene promoter region. Finally, we found that silencing either of these transcription factors in ESCs produced a decrease in Sod2 mRNA levels, indicating that these factors have a role in Sod2 gene modulation in ESCs.

In summary, we have presented a modest landscape of the transcriptional modulation of antioxidant defense components in pluripotent stem cells and found that Sod2, which is critical for cellular defense against ROS and that may also play a role in pluripotency maintenance, is induced by both Oct4 and Nanog. We consider that these findings may contribute to the comprehension of the oxidative stress defense system in pluripotent stem cells, and we are currently studying Gsr and Sod1 genes regulation since their expression also decreased along differentiation and their promoters also contain putative binding sites for the pluripotency transcription factors Oct4, Sox2 and Nanog. We hope that these results will contribute to improve the knowledge of the mechanisms involved in the ability of stem cells to maintain an intact genome, critical for their future applications in the field of tissue engineering and cell therapy.

## Supporting Information

S1 Fig Expression level of genes involved in stress defense along *in vitro* differentiation in mouse pluripotent stem cells. **(A)** R1 ESC line **(B)** Ainv15 ESC line **(C)** iPSC-20 line. **(i)** Representative pictures of undifferentiated colonies (left panels), embryoid bodies (EB) obtained by *in vitro* hanging drop differentiation protocol (middle panels) and differentiated cells obtained after embryoid bodies’ attachment (right panels). Scale bars: 100 μm **(ii)** Pluripotent stem cells were subjected to the hanging drop protocol and gave rise to embryoid bodies that were attached to gelatin coated plates. RNA was extracted from undifferentiated cells (D0), and at days 4 (D4) and 7 (D7) after EBs’ attachment. mRNA levels were measured by RT-qPCR and relativized to D0, shown as a dashed line. Gene expression was normalized to Gapdh. Results are shown as mean ± SEM of two independent experiments. Oct4, Nanog and Sox2 modulation along differentiation is shown **(iii)**; Sod1, Sod2, Cat, Prdx1, Prdx2, Gpx1, Gpx4, Txn1, Txn2, Glrx1, Glrx2, Txnrd1, Txnrd2 and Gsr were analyzed.
(TIF)

S2 Fig Neural progenitor differentiation of 46C embryonic stem cell line. 46C ESC were subjected to neural precursor differentiation protocol for 6 days. **(A)** Representative pictures of day 6 of differentiation showing expression of GFP driven by Sox1 promoter, marker of neuroectoderm. Bright field, left panel; GFP, right panel. Scale bars: 200 μm. **(B)** RNA was extracted at days 0 (D0), 3 (D3) and 6 (D6) after the induction of differentiation and mRNA levels were measured by RT-qPCR. Gene expression of the neural differentiation markers Blbp and Nestin was normalized to the geometrical mean of Gapdh and Pgk1 expression and referred to D0. Results are shown as mean ± SEM of three independent experiments. Different letters indicate statistically significant differences between treatments (p < 0.05).
(TIF)

S1 Table Primer sequences. (DOCX)

S2 Table Putative binding sites for pluripotent stem cell specific transcription factors in promoter sequences of genes involved in stress defense. Analysis of gene promoter sequences range from transcription start site (+1) to 5 kbp upstream using the MatInspector software. The number of putative binding sites is indicated for each transcription factor (TF). * Composed binding site for Oct4, Sox2, Nanog, Tcf3 (Tcf7l1) and Sall4b in pluripotent cells. TF: Transcription Factor; Sod: Superoxide dismutase; Prdx: Peroxiredoxin; Gpx: Glutathione peroxidase; Cat: Catalase; Glrx: Glutaredoxin; Txn: Thioredoxin; Txnrd: Thioredoxin reductase; Gsr: Glutathione reductase.
(DOCX)

S3 Table Position of putative binding sites for pluripotent stem cell specific transcription factors in mouse promoter sequences of genes involved in stress defense. Analysis of gene promoter sequences range from transcription start site (+1) to 5 kbp upstream using the MatInspector software. * Composed binding site for Oct4, Sox2, Nanog, Tcf3 (Tcf7l1) and Sall4b in pluripotent cells. Sod: Superoxide dismutase; Prdx: Peroxiredoxin; Gpx: Glutathione peroxidase; Cat: Catalase; Glrx: Glutaredoxin; Txn: Thioredoxin; Txnrd: Thioredoxin reductase; Gsr: Glutathione reductase.
(XLSX)

## Abbreviations

Cat: Catalase
ESC: embryonic stem cell
Pgk1: Phosphoglycerate kinase 1
Glrx: Glutaredoxin
Gpx: Glutathione peroxidase
Gsr: Glutathione reductase
Gapdh: Glyceraldehyde-3-phosphate dehydrogenase
iPSC: induced Pluripotent Stem Cell
Prdx: Peroxiredoxin
ROS: reactive oxygen species
Sod: Superoxide dismutase
Txn: Thioredoxin
Txnrd: Thioredoxin reductase
TF: transcription factor
