# Supplementary figures and images for: Manganese Superoxide Dismutase Gene Expression Is Induced by Nanog and Oct4, Essential Pluripotent Stem Cells’ Transcription Factors
Source: PLoS One. 2015 Dec 7;10(12):e0144336. doi: 10.1371/journal.pone.0144336 (PMC4671669; doi:10.1371/journal.pone.0144336)

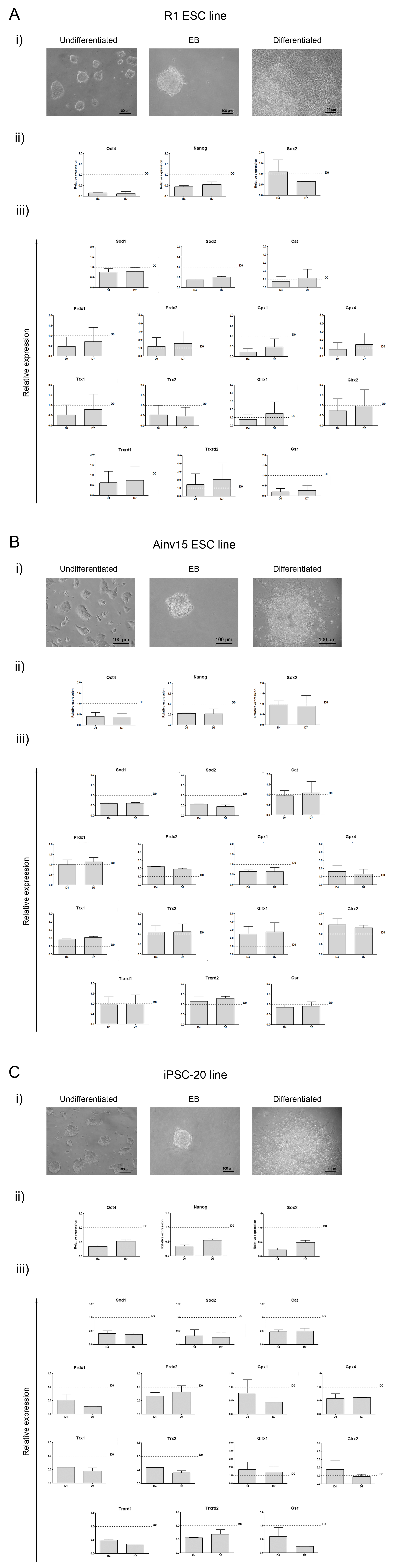

Supplement: S1 Fig — (A) R1 ESC line (B) Ainv15 ESC line (C) iPSC-20 line. (i) Representative pictures of undifferentiated colonies (left panels), embryoid bodies (EB) obtained by in vitro hanging drop differentiation protocol (middle panels) and differentiated cells obtained after embryoid bodies’ attachment (right panels). Scale bars: 100 μm (ii) Pluripotent stem cells were subjected to the hanging drop protocol and gave rise to embryoid bodies that were attached to gelatin coated plates. RNA was extracted from undifferentiated cells (D0), and at days 4 (D4) and 7 (D7) after EBs’ attachment. mRNA levels were measured by RT-qPCR and relativized to D0, shown as a dashed line. Gene expression was normalized to Gapdh. Results are shown as mean ± SEM of two independent experiments. Oct4, Nanog and Sox2 modulation along differentiation is shown (iii); Sod1, Sod2, Cat, Prdx1, Prdx2, Gpx1, Gpx4, Txn1, Txn2, Glrx1, Glrx2, Txnrd1, Txnrd2 and Gsr were analyzed. (TIF) [file pone.0144336.s001.tif]

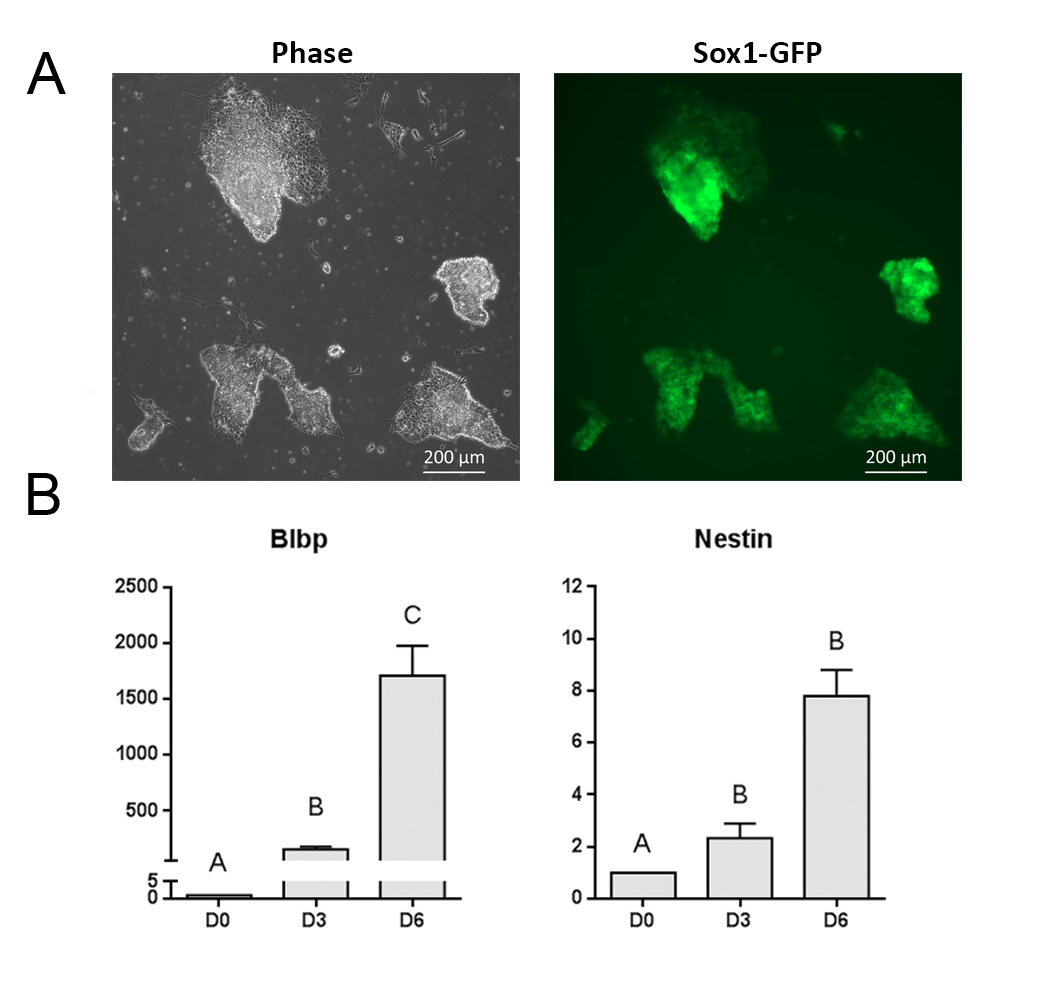

Supplement: S2 Fig — 46C ESC were subjected to neural precursor differentiation protocol for 6 days. (A) Representative pictures of day 6 of differentiation showing expression of GFP driven by Sox1 promoter, marker of neuroectoderm. Bright field, left panel; GFP, right panel. Scale bars: 200 μm. (B) RNA was extracted at days 0 (D0), 3 (D3) and 6 (D6) after the induction of differentiation and mRNA levels were measured by RT-qPCR. Gene expression of the neural differentiation markers Blbp and Nestin was normalized to the geometrical mean of Gapdh and Pgk1 expression and referred to D0. Results are shown as mean ± SEM of three independent experiments. Different letters indicate statistically significant differences between treatments (p < 0.05). (TIF) [file pone.0144336.s002.tif]
