# Supplementary material for: Manganese Superoxide Dismutase Gene Expression Is Induced by Nanog and Oct4, Essential Pluripotent Stem Cells’ Transcription Factors
Source: PLoS One. 2015 Dec 7;10(12):e0144336. doi: 10.1371/journal.pone.0144336 (PMC4671669; doi:10.1371/journal.pone.0144336)
Supplement: S1 Table — (DOCX) [file pone.0144336.s003.docx]

**S1 Table. Gene specific primers used in RTq-PCR**

| *Gene name* |  | *Primer sequence (5' a 3')* | *Product size (bp)* |
| --- | --- | --- | --- |
| Oct3/4 | Forward | TGACGGGAACAGAGGGAAAG | 254 |
|  | Reverse | TCAGCTTGGGCTAGAGAAGG |  |
| Nanog | Forward | AGGGTCTGCTACTGAGATGCTCTG | 364 |
|  | Reverse | CAACCACTGGTTTTTCTGCCACCG |  |
| Sox2 | Forward | CACAACTCGGAGATCAGCAA | 190 |
|  | Reverse | CTCCGGGAAGCGTGTACTTA |  |
| Gapdh | Forward | TGCCAAGGCTGTGGGCAAGG | 248 |
|  | Reverse | CGAAGGTGGAAGAGTGGG |  |
| Pgk1 | Forward | TGGGCAAGGATGTTCTGTTC | 242 |
|  | Reverse | TGCAGTCCCAAAAGCATCAT |  |
| Cat | Forward | ATACCTGTGAACTGTCCCTACCG | 283 |
|  | Reverse | GGTGGCCGGCAATGTTCTCAC |  |
| Txn1 | Forward | TGGTGAAGCTGATCGAGAGC | 118 |
|  | Reverse | GGCTTGATCATTTTGCAAGG |  |
| Txn2 | Forward | GGATGGACCTGACTTTCAAG | 138 |
|  | Reverse | ACCTTCCCGTGCTGCTTGGC |  |
| Glxn1 | Forward | GTTCATCAAGCCCACCTGCCC | 206 |
|  | Reverse | GATCACTGCATCCGCCTAT |  |
| Gpx4 | Forward | CGCTGTGCGCGCTCCATGC | 106 |
|  | Reverse | AGGCCACGTTGGTGACGATG |  |
| Sod1 | Forward | GTGTGCGTGCTGAAGGGCG | 237 |
|  | Reverse | GTCTCCAACATGCCTCTCTTC |  |
| Sod2 | Forward | AAGCACCACGCGGCCTACG | 119 |
|  | Reverse | CCATTGAACTTCAGTGCAGGCTG |  |
| Txnrd1 | Forward | TGTGTGAATGTGGGTTGCATACC | 381 |
|  | Reverse | GGAGAAAAGATCATCACTGC |  |
| Txnrd2 | Forward | GTGAAATCCTTGAACTGGGG | 220 |
|  | Reverse | CACTTGTGATTCCATATTCC |  |
| Gsr | Forward | GGGTGGCACTTGCGTGAATG | 167 |
|  | Reverse | GGCGGCTCACATAGGCATCCC |  |
| Prdx1 | Forward | CACCATTGCTCAGGATTATGG | 137 |
|  | Reverse | TCTCATCCACAGAGCGGCC |  |
| Prdx2 | Forward | GTGTCCTTCGCCAGATCAC | 412 |
|  | Reverse | CTAATACTTTATTGGTTTCC |  |
| Gpx1a | Forward | CGTGGACTGGTGGTGCTCGG | 235 |
|  | Reverse | GGTCGGTCATGAGCGCAGTG |  |
| Glrx2 | Forward | ACAGCACATCGTCGTTTTGG | 296 |
|  | Reverse | GTGAAGCCTGTGAGTGTCCG |  |
