# Supplementary material for: Manganese Superoxide Dismutase Gene Expression Is Induced by Nanog and Oct4, Essential Pluripotent Stem Cells’ Transcription Factors
Source: PLoS One. 2015 Dec 7;10(12):e0144336. doi: 10.1371/journal.pone.0144336 (PMC4671669; doi:10.1371/journal.pone.0144336)
Supplement: S2 Table — Analysis of gene promoter sequences range from transcription start site (+1) to 5 kbp upstream using the MatInspector software. The number of putative binding sites is indicated for each transcription factor (TF). * Composed binding site for Oct4, Sox2, Nanog, Tcf3 (Tcf7l1) and Sall4b in pluripotent cells. TF: Transcription Factor; Sod: Superoxide dismutase; Prdx: Peroxiredoxin; Gpx: Glutathione peroxidase; Cat: Catalase; Glrx: Glutaredoxin; Txn: Thioredoxin; Txnrd: Thioredoxin reductase; Gsr: Glutathione reductase. (DOCX) [file pone.0144336.s004.docx]

**S2 Table. Putative binding sites for pluripotent stem cell specific transcription factors in promoter sequences of genes involved in stress defense**

|  | *Gene promoter* | | | | | | | | | | | | | |
| --- | --- | --- | --- | --- | --- | --- | --- | --- | --- | --- | --- | --- | --- | --- |
| *TF* | Sod1 | Sod2 | Prdx1 | Prdx2 | Gpx1 | Gpx4 | Cat | Glrx1 | Glrx2 | Txn1 | Txn2 | Txnrd1 | Txnrd2 | Gsr |
| Oct4 | 5 | 8 | 13 | 0 | 5 | 4 | 1 | 9 | 5 | 6 | 8 | 10 | 8 | 8 |
| Nanog | 5 | 6 | 1 | 2 | 4 | 5 | 2 | 1 | 2 | 4 | 1 | 4 | 1 | 8 |
| Sox2 | 1 | 3 | 0 | 4 | 1 | 5 | 1 | 3 | 3 | 0 | 1 | 1 | 3 | 1 |
| Stem* | 2 | 2 | 0 | 3 | 3 | 3 | 2 | 1 | 4 | 6 | 1 | 2 | 1 | 1 |
